# Pan-Cancer Bioinformatics-Guided Evaluation of San-Huang-Xie-Xin-Tang Identifies Kidney Renal Clear Cell Carcinoma as a Potentially Responsive Cancer Type

**DOI:** 10.3390/ph19060936

**Published:** 2026-06-14

**Authors:** Syu-You Zuo, Yu-Pao Chou, Tai-Hsuan Hsu, Jan-Gowth Chang, Wen-Ling Chan

**Affiliations:** 1 Department of Bioinformatics and Medical Engineering, Asia University, Taichung 413, Taiwan; 110225001@live.asia.edu.tw (S.-Y.Z.); 111225001@live.asia.edu.tw (Y.-P.C.); 2 Department of Materials Science and Engineering, National Yang Ming Chiao Tung University, Hsinchu 30010, Taiwan; thhsu.dmbt@gmail.com; 3 Department of Pathology, Show Chwan Memorial Hospital, Changhua 542, Taiwan; jgchang99@gmail.com; 4 Department of Research and Development, Show Chwan Healthcare System, Changhua 542, Taiwan

**Keywords:** San-Huang-Xie-Xin-Tang, traditional Chinese medicine, bioinformatics, prognosis, pan-cancer analysis, systems pharmacology, pharmacogenomics

## Abstract

**Background/Objectives:** San-Huang-Xie-Xin-Tang (SHXXT) is a classical traditional Chinese herbal formula composed of *Coptis chinensis*, *Scutellaria baicalensis*, and *Rheum palmatum*, with documented anti-inflammatory and anticancer properties. Despite growing interest in its pharmacological potential, systematic evaluation of its gene regulatory effects across multiple cancer types remains limited. This study aimed to assess the prognostic relevance of SHXXT-regulated genes across pan-cancer contexts using publicly available transcriptomic and clinical datasets. **Methods:** Fifteen active compounds of SHXXT were identified from traditional Chinese medicine databases (Encyclopaedia of Traditional Chinese Medicine (ETCM) 2.0, Chinese Compound Medicine Database (ccTCM), and Integrated Traditional Chinese Medicine Database (ITCM)). Compound-induced gene expression profiles were obtained from MCF7-based transcriptomic perturbation data in the ITCM database and integrated with The Cancer Genome Atlas (TCGA) across 24 cancer types. Survival-associated genes were evaluated using Cox proportional hazards regression and Kaplan–Meier analysis. A weighted prognostic scoring framework, supported by normalization and sensitivity analyses, was developed to prioritize cancer types according to the concordance between SHXXT-induced gene regulation and favorable prognostic patterns. Functional enrichment analysis was performed using Annotation, Visualization, and Integrated Discovery (DAVID), and cancer-related genes were annotated using the OncoKB database. Complementary in vitro studies, including Annexin V/propidium iodide (PI) and MT-1 staining assays, were conducted in Hep3B cells using a Good Manufacturing Practice (GMP)-certified commercial SHXXT preparation. **Results:** SHXXT-regulated genes were significantly enriched in cancer-related pathways, particularly the PI3K–Akt and MAPK signaling pathways. Pan-cancer analysis revealed substantial heterogeneity in prognostic alignment across cancer types. Among the 24 cancer cohorts analyzed, kidney renal clear cell carcinoma (KIRC) achieved the highest prognostic alignment score within the proposed framework. In KIRC, several genes, including *PIK3CA*, *PIK3CB*, *KRAS*, and *RAF1*, remained significantly associated with favorable prognostic alignment after multivariable adjustment. Pathway enrichment analysis further identified PI3K–Akt and MAPK signaling as the most significantly represented pathways among favorably aligned genes. In contrast, hepatocellular carcinoma exhibited a relatively low prognostic alignment score, consistent with in vitro observations indicating predominantly non-selective cytotoxic stress rather than cancer-specific therapeutic activity. **Conclusions:** SHXXT-regulated genes exhibited marked heterogeneity across cancer types, with KIRC was consistently prioritized as the top-ranked cancer type across multiple analytical scenarios, suggesting a strong concordance between SHXXT-associated gene regulation and favorable prognostic signatures. These findings represent computational predictions derived from transcriptomic and survival associations rather than direct evidence of therapeutic efficacy. The study provides a reproducible pan-cancer strategy for prioritizing candidate cancer types for future mechanistic and experimental validation of traditional Chinese medicine formulations.

## 1. Introduction

Cancer remains one of the leading causes of mortality worldwide. According to the World Health Organization (WHO), nearly one-sixth of all global deaths in 2020 were attributable to cancer. Although mortality rates for certain cancers, such as leukemia, melanoma, and kidney cancer, have declined in recent years, the incidence of several major malignancies, including breast, pancreatic, and uterine cancers, continues to increase [1,2]. In particular, breast and prostate cancers have shown annual incidence increases of approximately 1–3%. The growing global cancer burden, coupled with challenges in early detection and limited oncology resources in many low- and middle-income countries, continues to place substantial pressure on healthcare systems [3]. Furthermore, the biological complexity and heterogeneity of cancer complicate therapeutic decision-making. Although surgery, chemotherapy, radiotherapy, targeted therapies, and immunotherapies have improved patient outcomes, these treatments often remain associated with significant toxicity, financial burden, and variable efficacy across patient populations [4,5]. Consequently, increasing attention has been directed toward complementary therapeutic approaches that may enhance treatment outcomes and improve quality of life.

Traditional Chinese medicine (TCM) has been used for centuries in cancer management and is increasingly recognized as a complementary approach in integrative oncology [6,7,8,9]. Among TCM modalities, Chinese herbal medicine (CHM) is particularly widely used in East Asian countries, including Taiwan, where many patients incorporate herbal formulations alongside conventional cancer treatments to alleviate treatment-related adverse effects and improve overall well-being.

San-Huang-Xie-Xin-Tang (SHXXT) is a classical traditional Chinese herbal formula composed of Coptis chinensis, Scutellaria baicalensis, and Rheum palmatum. Extensive pharmacological studies have demonstrated that SHXXT possesses diverse biological activities [10], including anti-inflammatory [11], neuroprotective [12,13], antioxidant [14], immunomodulatory [15], and anticancer properties [16,17,18]. Population-based evidence from Taiwan’s National Health Insurance Database further indicated that SHXXT use was associated with a reduction in breast cancer-related mortality [19].

Several bioactive constituents of SHXXT, such as berberine [20,21,22,23,24], baicalein [25,26,27,28], and emodin [29,30,31,32,33], have also been extensively investigated for their anticancer potential. Experimental studies have shown that these compounds can inhibit tumor growth, induce apoptotic cell death, and attenuate cancer cell invasion and metastasis through the regulation of multiple signaling pathways involved in cell proliferation, survival, and tumor progression. Despite these promising findings, the molecular mechanisms and prognostic relevance of SHXXT-associated gene regulation across different cancer types remain incompletely understood.

Most previous studies have focused on individual compounds, specific cancer types, or network pharmacology-based predictions [34]. While conventional network pharmacology approaches provide valuable insights into compound–target interactions, they often rely primarily on curated interaction databases and do not directly incorporate transcriptomic perturbation responses or clinical outcome information. Consequently, the prognostic implications of SHXXT-associated gene regulation across diverse cancer contexts remain largely unexplored.

Recent advances in large-scale cancer genomics resources, particularly The Cancer Genome Atlas (TCGA), provide an unprecedented opportunity to investigate the relationship between gene regulation and clinical outcomes across multiple cancer types. Leveraging these resources, we developed a pan-cancer analytical framework that integrates SHXXT-induced transcriptomic perturbation signatures with cancer-specific gene expression and survival data. A weighted prognostic scoring system was further established to evaluate the concordance between SHXXT-associated gene regulation and favorable prognostic patterns across 24 cancer types. Unlike conventional network pharmacology studies, this framework incorporates experimentally derived transcriptomic perturbation profiles obtained from a standardized pharmacogenomic platform, survival-associated gene prioritization, and prognostic directionality scoring to identify cancer types with the strongest prognostic alignment.

Using this approach, we systematically evaluated the potential prognostic relevance of SHXXT-regulated genes across multiple cancer types and identified kidney renal clear cell carcinoma (KIRC) as the highest-ranked cancer type within the proposed framework. Importantly, this ranking should be interpreted as a computational prioritization result rather than evidence of therapeutic efficacy.

It should be noted that all transcriptomic perturbation profiles analyzed in this study were generated using the MCF7 breast cancer cell line available in the Integrated Traditional Chinese Medicine (ITCM) database (http://itcm.biotcm.net/, accessed on 19 March 2024). Therefore, the resulting regulatory signatures may not fully capture tissue-specific responses across different cancer types. Because transcriptional responses may vary substantially among tissue types, the resulting findings should be regarded as hypothesis-generating and require validation in disease-relevant experimental systems.

## 2. Results

### 2.1. Selection and Composition Analysis of SHXXT Constituents

SHXXT is composed of three medicinal herbs: *Coptis chinensis*, *Scutellaria baicalensis*, and *Rheum palmatum* (rhubarb). To characterize the major chemical constituents of SHXXT, compound composition data were obtained from the Chinese Compound Medicine Database (ccTCM v.13) [35]. Constituents present at proportions exceeding 0.5% were initially considered representative components of each herb. To improve biological relevance and ensure compatibility with downstream transcriptomic analyses, additional constituents were included based on published evidence of pharmacological activity and the availability of corresponding perturbation profiles in the ITCM. The selected compounds were intended to represent the major bioactive constituents of SHXXT and to facilitate the investigation of their transcriptomic regulatory effects rather than to imply direct therapeutic efficacy.

Using these criteria, six compounds were selected according to their relative abundance in ccTCM, while an additional nine compounds were incorporated based on literature support and ITCM annotations. In total, 15 active compounds were retained for subsequent analyses, comprising three compounds from *C. chinensis*, five from *S. baicalensis*, and seven from rhubarb (Table 1).

Among these constituents, berberine, baicalin, and catechin represented some of the most abundant compounds identified in their respective herbs, whereas several lower-abundance constituents, including emodin, chrysophanol, and wogonin, were retained because of their documented biological activities and availability within the ITCM perturbation dataset. Collectively, these 15 compounds represent the predominant SHXXT-associated constituents currently available for transcriptomic perturbation analysis and formed the basis for subsequent pan-cancer investigations.

### 2.2. Analysis of Gene Regulation by SHXXT Active Components

**Figure 1:**
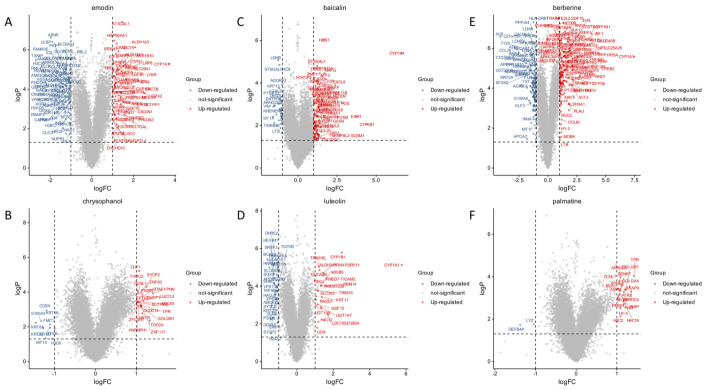
Transcriptomic perturbation profiles induced by representative SHXXT constituents. Volcano plots illustrating differential gene expression in MCF7 cells following treatment with six SHXXT compounds: (**A**) emodin, (**B**) chrysophanol, (**C**) baicalin, (**D**) luteolin, (**E**) berberine, and (**F**) palmatine. Red and blue dots represent significantly upregulated and downregulated genes, respectively, whereas gray dots indicate non-significant genes. Differentially expressed genes were defined using the thresholds of |log_2_FC| > 1 and FDR < 0.05.

Transcriptomic perturbation profiles for the 15 selected SHXXT constituents were obtained from the ITCM database, which provides gene expression data generated using the MCF7 breast cancer cell line. Each compound was evaluated using three biological replicates, enabling statistical assessment of compound-induced transcriptional changes. Differential expression analyses were performed separately for each compound, and genes with a Benjamini–Hochberg false discovery rate (FDR) < 0.05 were considered significantly regulated. Among the 15 compounds analyzed, six compounds—emodin, chrysophanol, baicalin, luteolin, berberine, and palmatine—exhibited extensive transcriptomic perturbation effects and regulated large numbers of genes (Figure 1A–F). Notably, these highly active compounds originated from all three constituent herbs of SHXXT, indicating that each herbal component contributed to the overall regulatory landscape of the formula.

Representative volcano plots illustrating the transcriptional responses induced by these six compounds are shown in Figure 1. Both upregulated and downregulated genes were observed across all compounds, although the magnitude and distribution of transcriptional changes varied considerably among individual constituents. These findings suggest that SHXXT contains multiple bioactive compounds capable of modulating diverse gene regulatory networks and provide a molecular basis for subsequent pathway enrichment and pan-cancer analyses.

Following integration of the transcriptomic perturbation results from all 15 SHXXT constituents and removal of duplicate entries, a total of 12,121 unique genes were identified as significantly regulated and retained for subsequent analyses.

**Figure 2:**
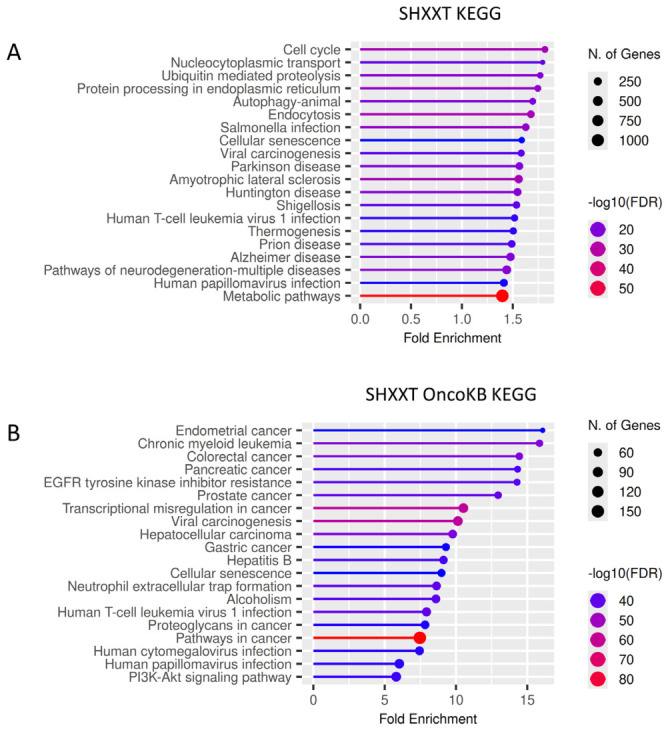
KEGG pathway enrichment analysis of SHXXT-regulated genes. Bubble plots illustrating significantly enriched KEGG pathways among (**A**) all SHXXT-regulated genes (n = 12,121) and (**B**) SHXXT-regulated genes overlapping with the OncoKB cancer gene set. Bubble size represents the number of genes associated with each pathway, color intensity indicates statistical significance (−log_10_ FDR), and the *X*-axis denotes fold enrichment. Pathways are ranked according to enrichment significance.

To explore the biological functions associated with these SHXXT-regulated genes, Kyoto Encyclopedia of Genes and Genomes (KEGG) pathway enrichment analysis was performed. As shown in Figure 2A, the significantly enriched pathways included cell cycle regulation, ubiquitin-mediated proteolysis, protein processing in the endoplasmic reticulum, autophagy, and multiple metabolic pathways. These pathways exhibited substantial fold enrichment and are known to play important roles in cellular homeostasis, proliferation, and stress responses.

To further investigate potential cancer-related associations, the 12,121 SHXXT-regulated genes were intersected with the OncoKB cancer gene set (https://www.oncokb.org/, accessed on 28 January 2024), comprising 889 curated cancer-associated genes (Figure 2B). Subsequent KEGG enrichment analysis of the overlapping gene set revealed significant enrichment of multiple cancer-related pathways, including endometrial cancer, chronic myeloid leukemia, colorectal cancer, pancreatic cancer, and the PI3K–Akt signaling pathway. These findings suggest that SHXXT-regulated genes are closely associated with biological processes and signaling networks relevant to cancer development and progression.

Notably, the enrichment of the PI3K–Akt signaling pathway is consistent with previous studies reporting the involvement of SHXXT constituents in the regulation of cell survival, proliferation, and tumor-associated signaling pathways. Collectively, these findings provide transcriptomic evidence that SHXXT-associated compounds preferentially modulate biological pathways involved in cancer-related cellular processes, supporting the rationale for subsequent pan-cancer prognostic analyses.

### 2.3. Pan-Cancer Transcriptomic Analysis and Identification of Prognostic Genes

To investigate the potential prognostic relevance of SHXXT-regulated genes across diverse cancer contexts, transcriptomic and clinical data from 24 cancer types were obtained from TCGA. The analyzed cohorts included both primary tumor and solid tissue normal samples, encompassing a broad spectrum of malignancies with varying sample sizes and clinical characteristics (Table 2).

Univariate Cox proportional hazards regression analysis was performed for each cancer type to identify genes associated with overall survival. Following multiple-testing correction using the Benjamini–Hochberg procedure, genes with an FDR < 0.05 were defined as prognostic genes and retained for subsequent analyses. These prognostic gene sets were then integrated with SHXXT-regulated transcriptional signatures to enable pan-cancer comparisons.

The resulting datasets provided a framework for evaluating the concordance between SHXXT-associated gene regulation and survival-related expression patterns across different cancer types. By combining transcriptomic perturbation profiles with cancer-specific prognostic information, we systematically assessed whether SHXXT-regulated genes were associated with favorable or unfavorable survival outcomes within individual tumor types.

As summarized in Table 2, the 24 TCGA cohorts included 8,489 tumor samples and 740 normal tissue samples. Cohort sizes varied substantially across cancer types, ranging from 35 tumor samples in cholangiocarcinoma (CHOL) to 1,111 tumor samples in breast carcinoma (BRCA), thereby providing a diverse pan-cancer landscape for subsequent prognostic analyses.

The analyzed cohorts represented a wide range of cancer types and sample sizes, providing a comprehensive dataset for evaluating the prognostic relevance of SHXXT-associated transcriptional regulation across multiple malignancies.

### 2.4. Prediction of SHXXT Regulatory Effects on Cancer Prognosis

To systematically evaluate the potential prognostic relevance of SHXXT-associated transcriptional regulation across multiple cancer types, a pan-cancer prognostic scoring framework was established by integrating SHXXT-induced gene expression changes with cancer-specific transcriptomic and survival data. The framework quantified the degree of concordance between SHXXT-regulated genes and favorable or unfavorable prognostic expression patterns identified within each TCGA cohort.

#### Prognostic Scoring and Robustness Assessment

The prognostic scoring framework incorporated both the direction and magnitude of survival associations, assigning greater weight to genes with stronger prognostic effects. The resulting scores were aggregated at the cancer-type level to estimate the overall alignment between SHXXT-associated transcriptional regulation and cancer-specific prognostic signatures.

Substantial heterogeneity was observed across the 24 cancer types analyzed (Table 3). Notably, kidney renal clear cell carcinoma (KIRC) achieved the highest total prognostic score (2358), markedly exceeding all other cancer types. Uterine corpus endometrioid carcinoma (UCEC), breast carcinoma (BRCA), glioblastoma multiforme (GBM), and cholangiocarcinoma (CHOL) ranked among the next highest-scoring cohorts. These findings indicate that the extent of favorable prognostic alignment varied considerably across cancer types.

Importantly, cancer types with a large number of favorable prognostic genes did not necessarily achieve the highest overall scores, emphasizing the contribution of both prognostic directionality and weighting magnitude to the final ranking. Therefore, the resulting rankings reflect the relative concordance between SHXXT-regulated genes and cancer-specific prognostic signatures rather than direct evidence of therapeutic efficacy.

To assess the robustness of the scoring framework, two complementary validation analyses were performed. First, score normalization was conducted to account for potential influences of cohort size and gene number. KIRC retained the highest overall ranking across all normalization strategies (rank sum = 5; Supplementary Table S1), indicating that the observed prioritization was not driven by sample size or the number of significant genes.

Second, sensitivity analyses were performed using three alternative weighting schemes. KIRC consistently remained the top-ranked cancer type under all tested conditions (Supplementary Table S2), demonstrating that the ranking was robust to variations in weighting parameters. In contrast, cancer types with limited normal tissue samples, including SKCM, THYM, and PCPG, exhibited greater ranking variability, suggesting increased sensitivity to cohort imbalance and warranting cautious interpretation.

Collectively, these results support the stability and robustness of the proposed prognostic scoring framework and identify KIRC as the highest-priority cancer type for subsequent investigation.

KIRC exhibited a total prognostic score that was approximately five-fold higher than that of the second-ranked cancer type (UCEC), highlighting its unique degree of prognostic concordance within the proposed framework.

### 2.5. Identification of KIRC as the Top-Ranked Cancer Type in Pan-Cancer Analysis

Because KIRC achieved the highest prognostic alignment score among the 24 analyzed cancer types and consistently retained the top ranking across normalization and sensitivity analyses, it was selected for further investigation.

**Figure 3:**
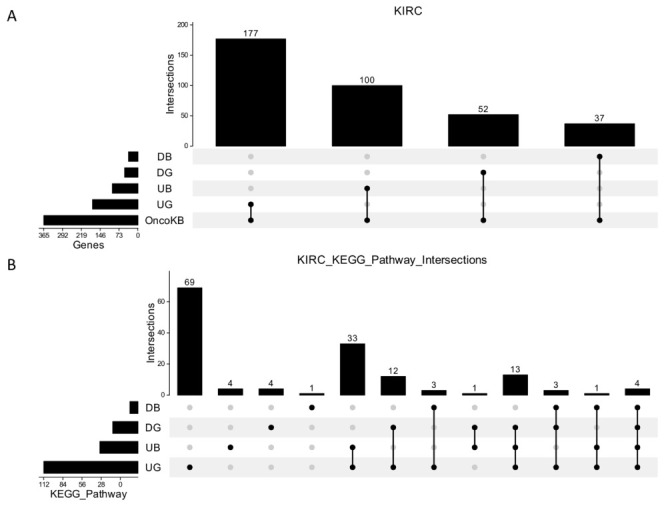
Intersection analysis of SHXXT-regulated genes and pathways in KIRC. (**A**) UpSet plot showing overlaps between OncoKB cancer genes and four prognostic regulatory groups. (**B**) UpSet plot showing overlaps among enriched KEGG pathways across the four regulatory groups. UG and DG represent concordant regulation associated with favorable prognostic directions, whereas UB and DB represent discordant regulation. Bar plots indicate the number of intersecting genes or pathways in each combination set.

To characterize the biological processes underlying the observed prognostic alignment, survival-associated genes identified in KIRC (FDR < 0.05) were subjected to KEGG pathway enrichment analysis using DAVID v6.8. The resulting pathways were subsequently integrated with the OncoKB cancer gene set and compared with SHXXT-regulated genes to identify cancer-relevant regulatory networks associated with KIRC (Figure 3A).

A substantial proportion of SHXXT-regulated genes overlapped with KIRC-associated transcriptomic signatures. Specifically, approximately 60% of SHXXT-regulated genes overlapped with either prognostically significant genes or differentially expressed genes (DEGs) identified in KIRC. Integration with the OncoKB database identified 366 cancer-associated genes distributed across four prognostic regulatory groups, including 229 genes within the concordant groups (UG and DG) and 137 genes within the discordant groups (UB and DB) (Figure 3A).

Pathway-level analysis demonstrated extensive overlap among biological pathways represented by the four regulatory groups (Figure 3B). Several canonical cancer-associated signaling pathways were enriched across multiple groups, including PI3K–Akt signaling, MAPK signaling, apoptosis, TNF signaling, and NF-κB-related pathways (Table 4).

Following multivariable Cox regression analyses adjusted for age and pathological stage, the concordant upregulated group (UG) contained several well-established cancer-associated genes, including *MAPK1*, *PIK3CA*, *PIK3CB*, *PIK3R1*, *KRAS*, *RAF1*, and *NRAS*. These genes were predominantly enriched in PI3K–Akt and MAPK signaling pathways. In contrast, the concordant downregulated group (DG) contained genes such as *PIK3R2*, *BAX*, and *RAC2*, which were associated with apoptosis and immune-related pathways. The discordant groups included genes involved in receptor tyrosine kinase signaling, TNF signaling, and NF-κB regulation, including *AKT1*, *ERBB2*, *TRAF2*, *NFKBIA*, and *BIRC3*.

Among all enriched pathways, PI3K–Akt signaling was significantly represented in the UG, UB, and DG groups, whereas MAPK signaling was enriched in the UG, UB, and DB groups. Notably, the strongest enrichment was consistently observed within the concordant upregulated group (UG), suggesting that SHXXT-associated transcriptional regulation preferentially aligned with favorable prognostic expression patterns in KIRC.

Collectively, these findings identify PI3K–Akt and MAPK signaling as major pathways associated with the observed prognostic alignment in KIRC and highlight several candidate genes that may contribute to the biological effects of SHXXT. Nevertheless, these observations are based on transcriptomic and survival associations and should be interpreted as hypothesis-generating rather than direct evidence of causal therapeutic activity.

### 2.6. Oncogene Expression and Mutation Status Exhibit Distinct Prognostic Associations Across Cancer Types

To further explore the biological significance of the cancer-associated genes identified in KIRC, representative oncogenes from the prognostically concordant regulatory groups were examined across multiple cancer types. Particular attention was given to *KRAS* and *BRAF* because of their established roles in tumorigenesis and their representation within the enriched signaling pathways identified in KIRC.

The analyses revealed that the prognostic implications of oncogene expression and mutation status varied substantially among cancer types. Importantly, these associations should not be interpreted as evidence that oncogenes function as tumor suppressors in specific malignancies. Rather, they reflect context-dependent relationships between gene activity and clinical outcomes.

**Figure 4:**
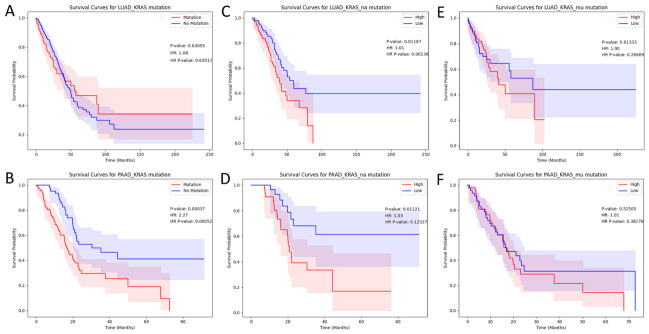
Prognostic associations of *KRAS* expression and mutation status in LUAD and PAAD. (**A**,**B**) Survival according to *KRAS* mutation status. (**C**,**D**) Survival according to *KRAS* expression in *KRAS* non-mutated patients (KRAS_na). (**E**,**F**) Survival according to *KRAS* expression in *KRAS*-mutated patients (KRAS_mu). Log-rank test *p*-values are shown.

For example, in lung adenocarcinoma (LUAD) and pancreatic adenocarcinoma (PAAD), lower *KRAS* expression levels and the absence of *KRAS* mutations were significantly associated with improved overall survival (Figure 4). In PAAD, patients harboring wild-type *KRAS* exhibited a marked survival advantage compared with those carrying *KRAS* mutations, consistent with the well-established oncogenic role of *KRAS* in pancreatic cancer.

**Figure 5:**
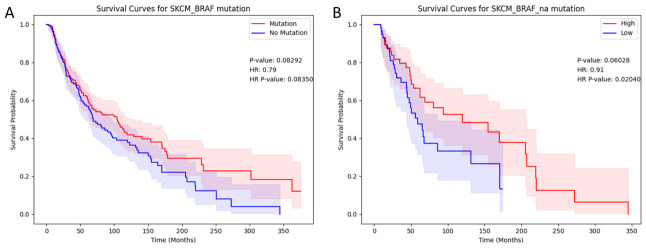
Prognostic associations of *BRAF* mutation and expression status in SKCM. (**A**) Overall survival according to *BRAF* mutation status. (**B**) Overall survival according to *BRAF* expression in patients without *BRAF* mutations (BRAF_na).

In contrast, skin cutaneous melanoma (SKCM) demonstrated a distinct prognostic pattern. Patients with *BRAF*-mutated tumors exhibited more favorable survival outcomes than those without detectable *BRAF* mutations (Figure 5A). Furthermore, among patients lacking *BRAF* mutations, higher *BRAF* expression levels showed a trend toward improved survival (Figure 5B).

**Figure 6:**
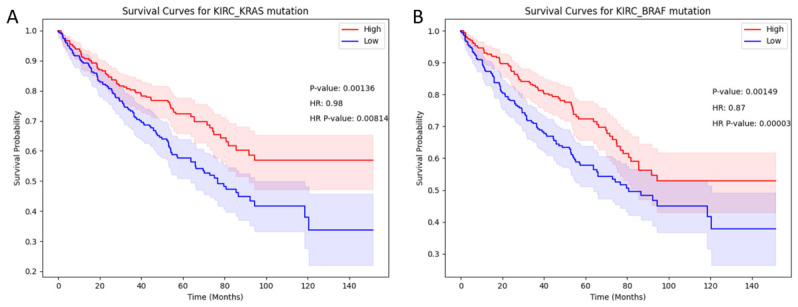
Kaplan–Meier survival analyses of representative oncogenes in KIRC. (**A**) Overall survival according to the *KRAS* mutation-associated gene signature. (**B**) Overall survival according to the *BRAF* mutation-associated gene signature.

Within KIRC, several oncogenes identified in the concordant regulatory groups, including *KRAS*, exhibited positive associations between higher expression levels and improved survival outcomes (Figure 6). These observations suggest that the prognostic significance of oncogene expression may depend on tumor-specific biological contexts, including molecular subtype composition, metabolic characteristics, immune microenvironment, and pathway dependency.

Collectively, these findings demonstrate substantial heterogeneity in the prognostic impact of oncogene expression and mutation status across cancer types. Therefore, the clinical interpretation of oncogene-associated biomarkers should be considered within the specific biological and pathological context of each malignancy rather than inferred solely from their canonical oncogenic functions.

### 2.7. Assessment of Cell Viability and Membrane Integrity by Annexin V/PI Staining

**Figure 7:**
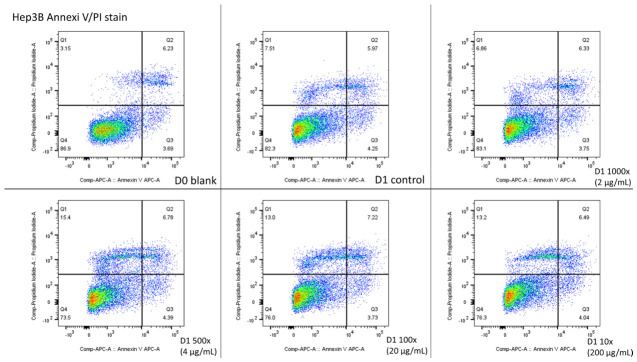
Flow cytometric analysis of Hep3B cells treated with SHXXT using Annexin V/propidium iodide (PI) staining. Cells were treated for 24 h with SHXXT at 200 µg/mL (10×), 20 µg/mL (100×), 4 µg/mL (500×), and 2 µg/mL (1000×). Increased PI-positive populations (Q1) were observed at higher concentrations, whereas apoptotic populations (Q2 and Q3) remained minimal. A progressive increase in the necrotic cell population was observed with increasing SHXXT concentrations. Data shown are representative of a single experiment.

To provide preliminary biological context for the transcriptomic findings, Hep3B cells were exposed to increasing concentrations of SHXXT and analyzed using Annexin V/propidium iodide (PI) staining (Figure 7). SHXXT treatment induced a concentration-dependent increase in membrane permeability, as evidenced by the progressive expansion of PI-positive cell populations at higher treatment concentrations.

Across all tested concentrations, apoptotic cell populations remained relatively limited, with no substantial increases observed in either early apoptotic (Q3) or late apoptotic (Q2) fractions. At the highest concentration tested (200 µg/mL), an atypical staining pattern suggestive of altered membrane integrity was observed. This phenomenon may reflect assay-related effects associated with extensive membrane damage rather than preservation of cellular viability.

**Figure 8:**
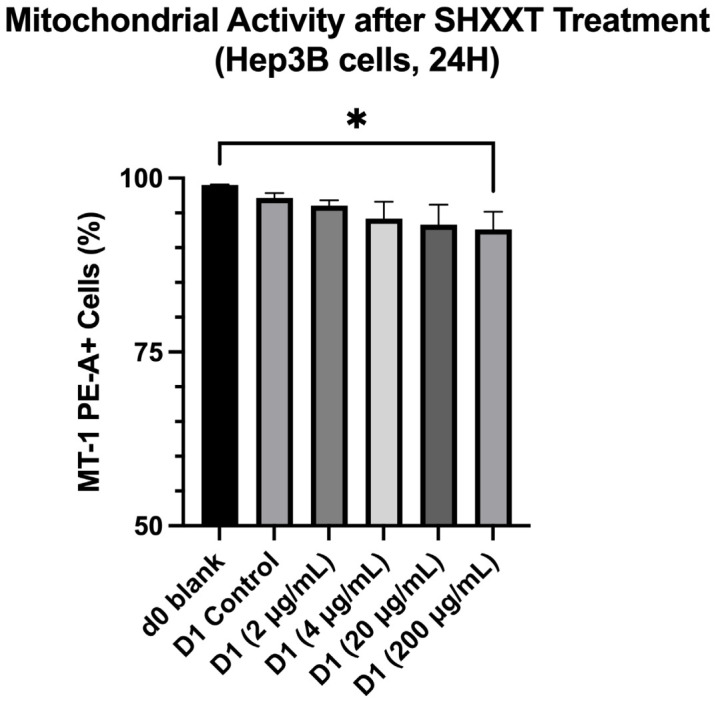
Mitochondrial activity of Hep3B cells following SHXXT treatment assessed by MT-1 staining. Bar graph showing the percentage of MT-1 PE-A+ cells after 24 h exposure to SHXXT. D0 blank represents untreated baseline cells, and D1 control represents vehicle-treated cells. Data are presented as mean ± SD from three independent biological replicates. Statistical significance was determined using Welch’s *t*-test. *: *p* < 0.05 compared with the blank control group (d0).

Consistent with the Annexin V/PI results, mitochondrial activity assessed by MT-1 staining demonstrated a concentration-dependent decline in PE-A-positive cell populations (Figure 8). Taken together, these findings suggest that high concentrations of SHXXT primarily induced cellular stress and loss of membrane integrity rather than activation of a predominant apoptotic program.

Importantly, these experiments were performed in Hep3B HCC cells and therefore should not be regarded as direct validation of the KIRC-associated predictions generated from the pan-cancer analysis. Instead, they were intended to provide preliminary biological context regarding the cellular consequences of high-concentration SHXXT exposure. Furthermore, the concentrations evaluated in these exploratory experiments may exceed physiologically achievable levels and therefore should not be interpreted as indicators of therapeutic efficacy or selectivity.

### 2.8. Assessment of Mitochondrial Function by MT-1 Staining

MT-1 staining was performed to evaluate mitochondrial function following SHXXT treatment in Hep3B cells. A concentration-dependent decline in PE-A+ cell proportions was observed across treatment groups, suggesting progressive impairment of mitochondrial function with increasing SHXXT exposure (Figure 8).

A statistically significant reduction in PE-A+ cells was detected when the highest SHXXT concentration (200 µg/mL) was compared with the untreated control group (D0; 92.9 ± 3.0% vs. 99.0 ± 0.1%, *p* = 0.0497, Welch’s *t*-test). Although no statistically significant differences were observed between the vehicle-treated control (D1) and individual SHXXT treatment groups (all *p* > 0.05), a consistent concentration-dependent downward trend was evident.

Representative MT-1 flow cytometry plots are provided in Supplementary Figure S1, and the corresponding quantitative measurements are summarized in Supplementary Table S3. Together, these supplementary data demonstrate the reproducibility of the observed trend across biological replicates.

The reduction in PE-A+ cell proportions at higher SHXXT concentrations is consistent with mitochondrial dysfunction and increased cellular stress. When considered alongside the Annexin V/PI staining results (Figure 7), these findings suggest that high concentrations of SHXXT primarily compromise cellular integrity and mitochondrial function rather than inducing a pronounced apoptotic response.

Nevertheless, these observations should be interpreted cautiously. The experiments were exploratory in nature and were performed exclusively in Hep3B cells. Consequently, the results should be regarded as supportive biological observations rather than direct validation of the KIRC-associated predictions generated by the pan-cancer analysis. Furthermore, the observed effects do not establish a specific molecular mechanism of action for SHXXT and require confirmation in disease-relevant experimental models.

## 3. Discussion

The present study developed a pan-cancer bioinformatics framework integrating TCM-derived transcriptomic perturbation profiles with large-scale cancer transcriptomic and survival datasets to evaluate the prognostic alignment of SHXXT-regulated genes across multiple cancer types. Among the 24 TCGA cancer cohorts analyzed, KIRC consistently achieved the highest prognostic alignment score and remained the top-ranked cancer type across multiple normalization strategies and alternative weighting schemes, suggesting that the prioritization was not driven by cohort size, gene number, or parameter selection. These findings support the robustness of the proposed framework and identify KIRC as a candidate cancer type for future mechanistic investigation.

Unlike conventional TCM network pharmacology studies, which primarily rely on predicted compound–target interactions and pathway enrichment analyses, the present framework incorporates experimentally derived transcriptomic perturbation signatures from the ITCM database together with cancer-specific differential expression and survival information. By integrating SHXXT-induced regulatory directionality with prognostic gene behavior, the proposed approach evaluates whether compound-associated transcriptional changes align with favorable or unfavorable survival-associated expression patterns. Consequently, the proposed framework provides a clinically relevant prioritization strategy for identifying cancer types that may warrant further mechanistic investigation.

One of the major findings of this study was the consistent enrichment of PI3K–Akt and MAPK signaling pathways among SHXXT-regulated genes and KIRC-associated prognostic signatures (Figure 2). These pathways are well-recognized regulators of cell proliferation, metabolism, survival, angiogenesis, and therapeutic resistance in renal cell carcinoma. Following multivariate Cox regression analyses adjusted for age and pathological stage, several genes within these pathways, including *KRAS*, *RAF1*, *PIK3CA*, *PIK3CB*, *PIK3R1*, *MAPK1*, and *NRAS*, remained within the concordant prognostic group. The persistence of these associations after adjustment for clinical variables suggests that the observed relationships are not solely attributable to basic clinicopathological confounders. Nevertheless, these findings should be interpreted as transcriptomic and prognostic associations rather than evidence of direct therapeutic modulation by SHXXT.

An interesting observation was that several genes traditionally classified as oncogenes, including *KRAS*, *RAF1*, and *PIK3CA*, were associated with favorable survival outcomes in KIRC. Importantly, these findings do not imply tumor-suppressive functions of these genes. Instead, they highlight the context-dependent nature of oncogene-associated prognostic relationships. Similar observations have been reported in several cancer types where elevated expression of canonical oncogenes may correlate with specific molecular subtypes, immune microenvironment characteristics, metabolic states, or treatment-responsive tumor phenotypes. Consistent with this interpretation, the prognostic associations of *KRAS* and *BRAF* varied markedly across cancer types in the present study. While lower *KRAS* expression and non-mutated status were associated with favorable outcomes in LUAD and PAAD (Figure 4), higher *KRAS* expression correlated with improved survival in KIRC (Figure 6). Likewise, *BRAF* mutation status demonstrated favorable associations in SKCM (Figure 5). These findings emphasize that prognostic significance should not be equated with biological function and that oncogene behavior remains highly dependent on tumor-specific contexts.

To provide biological context for the computational predictions, exploratory in vitro experiments were conducted using Hep3B cells. Annexin V/PI staining (Figure 7) demonstrated a concentration-dependent increase in membrane permeability and necrotic cell populations, whereas apoptotic populations remained limited. Similarly, MT-1 staining (Figure 8) revealed reduced mitochondrial activity at higher SHXXT concentrations. Collectively, these findings suggest that high concentrations of SHXXT predominantly induce non-specific cellular stress rather than a clearly defined apoptotic program. Importantly, these experiments were not designed to evaluate anticancer efficacy and should not be interpreted as validation of the KIRC-related computational predictions. Rather, they provide supportive evidence that SHXXT can perturb cellular metabolic processes and viability under experimental conditions. Notably, HCC ranked only eighth in the prognostic alignment analysis, further supporting the notion that the experimental observations in Hep3B cells do not directly correspond to the KIRC-specific findings identified through the pan-cancer framework.

The pathway enrichment analyses further supported the biological relevance of the identified candidate genes. After applying a more stringent enrichment threshold (FDR < 0.05), PI3K–Akt signaling remained significantly enriched within the OncoKB-filtered cancer gene set, together with several established cancer-associated pathways, including endometrial cancer, chronic myeloid leukemia, and colorectal cancer pathways. The persistence of these pathways under more stringent statistical criteria supports the stability of the pathway-level findings and suggests that the observed enrichment patterns are unlikely to be solely attributable to threshold selection.

### Limitations

Several limitations should be acknowledged. First, the study relied primarily on publicly available transcriptomic and clinical datasets and therefore represents a computational prioritization framework rather than a therapeutic efficacy study. Although normalization and sensitivity analyses demonstrated robust prioritization of KIRC, the scoring framework remains a biologically informed heuristic model rather than an optimized predictive algorithm. Future machine-learning-based approaches may further improve weighting selection and predictive performance.

Second, the transcriptomic perturbation signatures were derived exclusively from MCF7 breast cancer cells. Although the ITCM database provides a standardized pharmacogenomic resource, transcriptional responses to SHXXT compounds may vary substantially across tissues and cancer types. Consequently, the identified cancer rankings should be regarded as hypothesis-generating predictions that require validation in disease-relevant experimental systems.

Third, several TCGA cancer cohorts contained limited numbers of normal tissue samples, including SKCM, THYM, PCPG, and SARC. Although these datasets were retained to maximize pan-cancer coverage, differential expression estimates for these cancers may be more susceptible to sampling variability. This interpretation is supported by the greater ranking instability observed for some of these cohorts during sensitivity analyses.

Fourth, the current study did not model potential synergistic or antagonistic interactions among SHXXT components. The integrated regulatory signature was derived from transcriptomic perturbation profiles of individual compounds and therefore may not fully capture the pharmacological complexity of the complete herbal formulation.

Finally, no renal carcinoma cell lines were evaluated experimentally. The identification of KIRC as the highest-ranked cancer type therefore remains a computational prediction. Future validation using KIRC-derived models, including 786-O, Caki-1, A498, and patient-derived systems, will be necessary to determine whether the transcriptomic associations identified in this study translate into biologically meaningful therapeutic responses.

Overall, the present study provides a reproducible pan-cancer framework for integrating TCM-associated transcriptomic perturbation data with cancer survival information. While the findings do not establish therapeutic efficacy, they identify KIRC as a promising candidate for future mechanistic and translational studies investigating the potential role of SHXXT in renal cell carcinoma.

## 4. Materials and Methods

### 4.1. Research Approach

**Figure 9:**
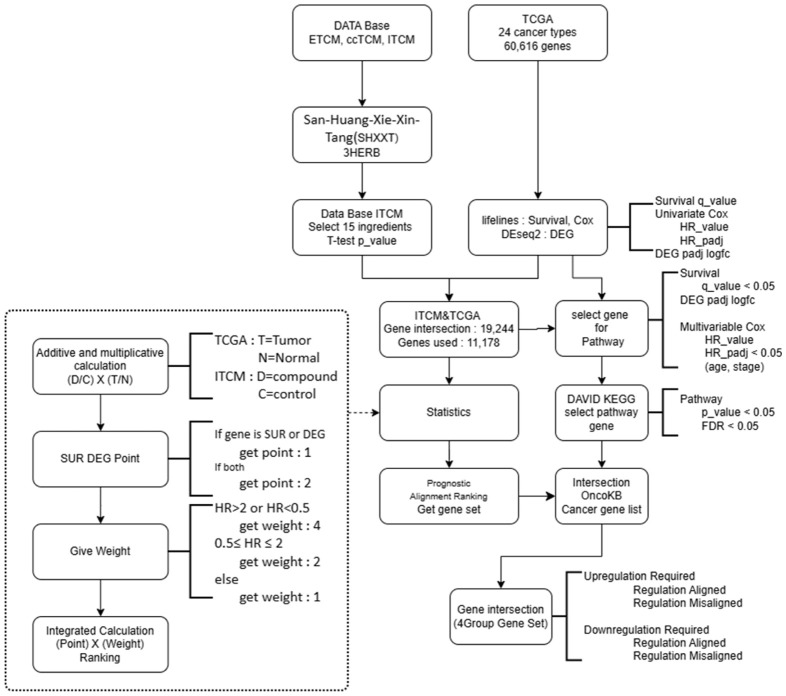
Integrated workflow of the prognostic alignment framework used to evaluate SHXXT-regulated genes across 24 cancer types. SHXXT-associated transcriptomic perturbation profiles obtained from ITCM were integrated with TCGA differential expression and survival analyses. A weighted scoring framework was used to assess the concordance between SHXXT-associated gene regulation and favorable prognostic expression patterns. Enriched pathways were identified using DAVID KEGG analysis and further annotated using OncoKB to identify cancer-associated genes and regulatory groups.

Figure 9 illustrates the overall analytical framework used to investigate the prognostic relevance of SHXXT-associated transcriptional regulation across multiple cancer types through the integration of TCM resources, pharmacogenomic perturbation profiles, cancer transcriptomic datasets, and survival analyses. The analytical framework consisted of five major components: (1) Data Collection and Resource Integration: Information regarding TCM herbs, formulas, bioactive compounds, target genes, and associated pathways was collected from ETCM2.0 [36,37], ccTCM v.1.3 [35] and the ITCM database [38]. In parallel, transcriptomic and clinical data from 24 TCGA cancer types comprising 60,616 genes were obtained for pan-cancer analyses. (2) Identification of SHXXT-Regulated Genes: Active compounds of SHXXT were selected according to their abundance in ccTCM and availability within the ITCM database. Gene expression profiles generated from compound-treated MCF7 cells were obtained from ITCM. Differential expression analyses were performed independently for each compound using three biological replicates. Genes with an FDR < 0.05 were considered significantly regulated and retained for downstream analyses. (3) Pan-Cancer Transcriptomic and Survival Analyses: Differential expression analyses were performed using DESeq2 (version 1.40.2), whereas survival analyses were conducted using the lifelines package (version 0.28.0) and Cox proportional hazards regression models. Genes with FDR-adjusted significance in either survival or differential expression analyses were designated as prognostic genes and integrated with SHXXT-regulated gene signatures. (4) Prognostic Alignment Scoring Framework: The regulatory effects of SHXXT were evaluated using the prognostic alignment scoring framework shown in Figure 9. For each gene, SHXXT-induced transcriptional regulation was compared with the prognostic direction inferred from TCGA analyses. Concordant regulation (alignment with favorable prognostic directions) received positive scores, whereas discordant regulation received negative scores. Weighted scores were assigned according to the magnitude of prognostic associations based on hazard ratios. The resulting scores were aggregated to generate cancer-type-specific prognostic alignment rankings. Normalization analyses and sensitivity analyses using alternative weighting schemes were subsequently performed to evaluate the robustness of the ranking framework. (5) Functional Enrichment and Cancer Gene Analyses: Genes identified through the scoring framework were subjected to KEGG pathway enrichment analysis using DAVID. Significantly enriched pathways were defined using FDR-adjusted *p*-values < 0.05. The resulting gene sets were further compared with the OncoKB cancer gene database (https://www.oncokb.org/) to identify cancer-associated genes. Based on the concordance between SHXXT-associated transcriptional regulation and prognostic expression patterns, genes were classified into four regulatory categories: concordant upregulated (UG), discordant upregulated (UB), concordant downregulated (DG), and discordant downregulated (DB) groups.

### 4.2. Selection of SHXXT Compounds and Data Sources

SHXXT was selected as the representative TCM formula for this study. Information regarding its constituent herbs, chemical components, and relative abundances was collected from the ETCM 2.0 [36,37] (http://www.tcmip.cn/ETCM2/front/, accessed on 19 March 2024), the ccTCM [35] (http://cctcm.org.cn/, accessed on 19 March 2024), and relevant literature sources.

To identify representative bioactive constituents of SHXXT, compounds with relative abundances exceeding 0.5% in the ccTCM database were initially selected. Additional compounds were included based on published evidence of pharmacological activity and the availability of corresponding transcriptomic perturbation profiles in the ITCM database [39]. Using these criteria, a total of 15 compounds were retained for subsequent analyses, representing the major bioactive constituents of SHXXT.

Transcriptomic perturbation data for the selected compounds were obtained from the ITCM database, which provides genome-wide gene expression profiles generated following compound treatment in MCF7 breast cancer cells. ETCM 2.0 and ccTCM were primarily used for compound identification and constituent abundance assessment, whereas ITCM served as the source of pharmacogenomic perturbation data. The integration of these complementary resources enabled the construction of SHXXT-associated transcriptional regulatory signatures for downstream analyses.

It should be noted that all perturbation profiles available in ITCM were generated using the MCF7 breast cancer cell line. Therefore, the resulting transcriptional signatures were used as a standardized pharmacogenomic resource and were not intended to represent tissue-specific biological responses. Consequently, subsequent analyses focused on the comparative evaluation of SHXXT-associated regulatory patterns across cancer types rather than direct inference of tissue-specific therapeutic effects.

### 4.3. Gene Expression Regulation Analysis

Transcriptomic perturbation profiles were obtained from the ITCM database [38] (https://itcm.idrblab.net/, accessed on 19 March 2024), which contains genome-wide gene expression data for 496 compounds and 20,030 genes generated using the MCF7 breast cancer cell line. Gene expression profiles corresponding to the 15 selected SHXXT-associated compounds were extracted for downstream analyses. For each compound, the ITCM dataset includes three biological replicates for both treatment and control conditions.

Differential expression analyses were performed independently for each compound using Student’s *t*-tests. To control for multiple testing across genome-wide analyses, *p*-values were adjusted using the Benjamini–Hochberg procedure. Genes with an FDR < 0.05 were considered significantly regulated and retained for subsequent analyses. Genes that did not meet this significance threshold for a given compound were excluded from compound-specific fold-change calculations.

For each significantly regulated gene, fold change (FC) was calculated as the ratio of mean expression in the treatment group to that in the control group. To summarize the overall regulatory effect of SHXXT across its constituent compounds, an integrated SHXXT FC was calculated using the geometric mean of statistically significant compound-specific fFCs:

$${FC}_{SHXXT}={\left(\prod_{i=1}^{n}{FC}_{i}\right)}^{1/n}$$

where ${FC}_{i}$ represents the FC induced by compound i, calculated as the mean expression of treated replicates divided by the mean expression of control replicates, and *n* denotes the number of compounds that significantly regulated the corresponding gene.

The geometric mean was selected because FCs represent multiplicative biological effects and may vary substantially among compounds. Compared with arithmetic averaging, the geometric mean reduces the influence of extreme values while preserving the overall direction and magnitude of transcriptional regulation. Only statistically significant compound-gene associations were included in the calculation to minimize signal dilution and improve biological interpretability.

Because all perturbation profiles available in ITCM were generated using the MCF7 breast cancer cell line, the resulting regulatory signatures were treated as standardized pharmacogenomic perturbation profiles rather than tissue-specific biological responses. Consequently, the identified regulatory patterns should be interpreted as hypothesis-generating and require validation in disease-relevant experimental systems.

### 4.4. Pan-Cancer Transcriptomic and Prognostic Gene Analyses

Transcriptomic and clinical data from 24 TCGA cancer cohorts were obtained and used for differential expression and survival analyses. TCGA RNA-seq datasets and ITCM transcriptomic perturbation profiles were analyzed independently within their respective platforms prior to integration. To minimize platform-specific systematic bias, integration was based on regulatory directionality, differential expression status, and prognostic associations rather than direct comparisons of raw expression values.

Differential expression analyses were performed separately for each cancer type using DESeq2 (version 1.40.2) in R (version 4.5.3; R Core Team, 2024, Vienna, Austria). Genes with an absolute log_2_ fold change (|log_2_FC|) > 1 and a Benjamini–Hochberg FDR < 0.05 were classified as DEGs.

Survival analyses were conducted using the Python (version 3.10) lifelines package (version 0.28.0) and corresponding clinical survival data obtained from the University of California, Santa Cruz (UCSC) Xena platform (https://xenabrowser.net/, accessed on 28 January 2024). Univariate Cox proportional hazards regression analyses were performed for all analyzed genes within each cancer cohort. To control for multiple testing, *p*-values were adjusted using the Benjamini–Hochberg procedure. Genes with an FDR < 0.05 were considered prognostically significant and retained for downstream analyses.

Hazard ratios (HRs) were used to determine the direction of prognostic associations. An HR < 1 indicated that higher gene expression was associated with improved survival, whereas an HR > 1 indicated that higher gene expression was associated with poorer survival outcomes.

Genes that did not meet the predefined survival significance threshold were retained within the prognostic alignment framework if they satisfied differential expression criteria. However, these genes were not considered independent evidence of prognostic relevance and were used only to characterize transcriptional regulatory patterns.

For KIRC, multivariable Cox proportional hazards regression analyses were additionally performed to evaluate whether candidate prognostic associations remained significant after adjustment for major clinical variables. Age at diagnosis was included as a continuous covariate, whereas pathological stage was treated as a categorical covariate. Genes that remained significant following multivariable adjustment were subsequently incorporated into KIRC-specific gene prioritization and pathway enrichment analyses. The complete lists of differentially expressed genes, prognostically significant genes, and corresponding FDR-adjusted statistics generated from the TCGA analyses are available upon reasonable request.

### 4.5. Assessment of SHXXT-Associated Regulatory Effects on Cancer Gene Expression

To evaluate the potential impact of SHXXT-associated transcriptional regulation within different cancer contexts, transcriptomic perturbation signatures derived from ITCM were integrated with cancer-specific gene expression profiles obtained from TCGA. Because the two datasets originated from independent experimental platforms, integration was performed using relative expression changes rather than raw expression values. For each gene, an integrated fold difference was calculated as:

$$\mathrm{Fold}\;\mathrm{Difference}\;=\;\frac{T}{N}\times \frac{D}{C}$$

where *T* and *N* represent the mean gene expression levels in tumor and normal tissues, respectively, and *D* and *C* represent the mean gene expression levels in SHXXT-treated and control samples, respectively.

The tumor-to-normal ratio (*T*/*N*) was used to characterize cancer-associated expression patterns, whereas the treatment-to-control ratio (*D*/*C*) represented SHXXT-induced transcriptional regulation. Multiplication of these two ratios enabled assessment of whether SHXXT-associated regulation would theoretically reinforce or counteract the expression patterns observed in a given cancer type.

Based on the direction of regulation and the corresponding prognostic associations identified from TCGA survival analyses, genes were classified into four prognostic regulatory groups:

UG (Upregulated-Good): SHXXT-induced upregulation aligned with a favorable prognostic expression pattern.
UB (Upregulated-Bad): SHXXT-induced upregulation aligned with an unfavorable prognostic expression pattern.
DG (Downregulated-Good): SHXXT-induced downregulation aligned with a favorable prognostic expression pattern.
DB (Downregulated-Bad): SHXXT-induced downregulation aligned with an unfavorable prognostic expression pattern.

Genes classified as UG or DG were considered concordant with favorable prognostic directions, whereas genes classified as UB or DB were considered discordant. These classifications formed the basis for the subsequent prognostic alignment scoring framework and pathway enrichment analyses. The resulting classifications were subsequently weighted according to the magnitude of prognostic associations and incorporated into the cancer-specific prognostic alignment scoring framework described in Section 4.6.

### 4.6. Prognostic Alignment Weighting and Scoring Framework

To quantify the degree of concordance between SHXXT-associated transcriptional regulation and cancer-specific prognostic expression patterns, a weighted scoring framework was developed based on HRs derived from Cox proportional hazards regression analyses. Genes were assigned prognostic weights according to the magnitude and statistical significance of their survival associations:

HR > 2 or HR < 0.5: Weight = 4 (strong prognostic association).
0.5 ≤ HR ≤ 2 and FDR < 0.05: Weight = 2 (moderate prognostic association).
FDR ≥ 0.05: Weight = 1 (non-significant association).

The weighting scheme was designed as a biologically informed heuristic framework rather than a predictive machine learning model. Genes exhibiting stronger survival associations were assigned larger weights and therefore contributed more substantially to the overall cancer-specific prognostic alignment score.

For each gene, prognostic alignment was determined by comparing the direction of SHXXT-associated transcriptional regulation with the favorable prognostic direction inferred from TCGA survival analyses. Concordant regulation, defined as SHXXT-associated regulation that aligned with favorable prognostic expression patterns, received a positive score equal to the assigned weight. Conversely, discordant regulation received a negative score of the same magnitude.

For example, a gene exhibiting higher expression in tumor tissue than in normal tissue (*T*/*N* > 1) and a significant favorable prognostic association (HR < 0.5, FDR < 0.05) was considered to have a favorable prognostic direction characterized by increased expression. If SHXXT further increased the expression of this gene, the resulting regulatory effect was classified as concordant and assigned a score of +4. In contrast, SHXXT-induced downregulation of the same gene would be classified as discordant and assigned a score of −4.

To evaluate the robustness of the scoring framework, score normalization and sensitivity analyses were performed. Two normalization strategies were applied to account for potential influences of gene number and cohort size:

$$S_{gene}=\frac{S_{total}}{N_{sig}}$$

The score per 100 tumor samples was calculated as:

$$S_{sample}=\frac{S_{total}}{N_{tumor}}\times 100$$

where $S_{total}$ is the total prognostic score, $N_{sig}$ is the number of genes meeting significance thresholds in either survival or differential expression analysis, and $N_{tumor}$ denotes the number of tumor samples within the corresponding TCGA cohort.

In addition, sensitivity analyses were conducted using three alternative weighting schemes: (0, 1, 2), (1, 2, 3), and (0, 2, 4) for non-significant, moderate-risk, and strong-risk categories, respectively. The resulting rankings were subsequently compared to evaluate the stability of the prognostic alignment framework under different weighting conditions.

### 4.7. Pathway Enrichment Analysis

Pathway enrichment analysis was performed using the DAVID v6.8 platform https://davidbioinformatics.nih.gov/, accessed on 28 January 2024 [40,41] to identify KEGG pathways associated with SHXXT-regulated and prognostically relevant genes (FDR < 0.05). Genes identified through TCGA differential expression and survival analyses were integrated with SHXXT-associated genes exhibiting prognostic alignment with SHXXT-associated regulation.

KEGG pathway enrichment analyses were conducted separately for the resulting gene sets. Pathways with Benjamini–Hochberg FDR-adjusted *p*-values < 0.05 were considered significantly enriched. The enriched pathways were subsequently used to characterize biological processes and signaling networks potentially associated with cancer progression, prognosis, and SHXXT-related transcriptional regulation.

### 4.8. Composition and Preparation of SHXXT

SHXXT is a traditional Chinese herbal formula composed of three medicinal herbs. In this study, a commercially available SHXXT extract granule product was used. The product was manufactured under Good Manufacturing Practice (GMP) standards and purchased from Sheng Foong Pharmaceutical Co., Ltd. (Sheng Foong Co., Ltd., Taipei, Taiwan). According to the manufacturer’s label information, each 9 g of the SHXXT granules contains authenticated crude herbal materials derived from Rheum palmatum L. (Radix et Rhizoma Rhei; Da Huang, 12.0 g), Coptis chinensis Franch. (Rhizoma Coptidis; Huang Lian, 6.0 g), and Scutellaria baicalensis Georgi (Radix Scutellariae; Huang Qin, 6.0 g).

The herbal materials were decocted and concentrated to yield 4.5 g of dry herbal extract, corresponding to a crude drug–to–extract ratio of 24:4.5 (5.33:1). The final granule formulation additionally contained 4.5 g of corn starch as an excipient to facilitate granulation and dosage consistency.

The product name is “San Hwang Shieh Shin Tang Extract Granule”, product code E109, with a total package weight of 200 g. The product is registered with the Taiwan Food and Drug Administration (TFDA; license no. 042418) and labeled as Made in Taiwan. For in vitro experiments, SHXXT granules were dissolved in sterile distilled water, filtered through a 0.22 μm Millex-GS syringe filter (Cat. No. SLGS033, Merck Millipore, Burlington, MA, USA), and freshly prepared to the indicated working concentrations prior to cell treatment. All experiments were performed using freshly prepared SHXXT solutions.

### 4.9. Quality Control and Safety Assessment of SHXXT

The commercial SHXXT extract granules used in this study were manufactured under Good Manufacturing Practice (GMP) conditions by a TFDA-licensed pharmaceutical company in Taiwan. According to the manufacturer, each production batch is accompanied by a Certificate of Analysis (COA) as part of routine quality assurance and regulatory compliance.

According to the manufacturer-provided COA, quality control testing included assessments of heavy metal contamination (lead, arsenic, cadmium, and mercury), pesticide residues, microbial limits, and extract specifications. All measured parameters were reported to comply with TFDA regulations and relevant pharmacopeial standards for traditional Chinese medicine products marketed in Taiwan. The COA additionally documented batch identity, physical appearance, and manufacturing specifications to ensure production consistency. To minimize potential variability, all experiments in the present study were performed using material obtained from a single manufacturing batch.

Independent analytical verification of heavy metals, pesticide residues, or phytochemical composition was not performed as part of the current investigation. Nevertheless, the use of a GMP-certified, TFDA-approved product accompanied by an official COA provides a recognized regulatory framework for quality assurance and product safety. Because the present study was limited to in vitro cell-based experiments, no direct human or animal exposure occurred. Future studies will incorporate independent chemical profiling, quantitative phytochemical analyses, and contaminant assessments to further strengthen product characterization, quality control, and experimental reproducibility.

### 4.10. Cell Line Culture

HCC Cell line Hep3B (HB-8064) was obtained from American Type Culture Collection (ATCC) (Manassas, VA, USA). Cell line authentication was performed by ATCC using short tandem repeat (STR) profiling prior to distribution. Upon receipt, cells were expanded, cryopreserved, and used within 10 passages to minimize potential genetic drift. Hep3B cells were routinely monitored for morphological characteristics and growth behavior during culture. Mycoplasma contamination was assessed using a PCR-based detection assay, and all cells were confirmed to be mycoplasma-free before use in experiments. Cells were cultured in Dulbecco’s modified Eagle medium/Nutrient Mixture F-12 (#11320033, Thermo Fisher Scientific, Inc., Waltham, MA, USA) supplemented with 10% fetal bovine serum (Gemini Bio-Products, Inc., West Sacramento, CA, USA) under standard conditions (5% CO_2_, 37 °C) over a week for stabilization. Then the cells were cryopreserved in KM Banker (#KOJ-16092005, Kohjin Bio Co., Ltd., Sakado city, Saitama, Japan) for experiments as described below. For long-term storage, cells were cryopreserved in KM Banker cryopreservation medium (Cat. No. KOJ-16092005, Kohjin Bio Co., Ltd., Saitama, Japan), according to the manufacturer’s instructions. Hep3B cells were used solely for exploratory assessment of SHXXT-induced cellular responses and were not intended as disease-specific validation models for KIRC.

### 4.11. Assessment of Cell Viability, Membrane Integrity, and Mitochondrial Function

To provide supportive experimental context for the bioinformatics analyses, the effects of SHXXT on Hep3B cell viability and physiological status were evaluated using flow cytometry-based assays. SHXXT granules were dissolved in sterile phosphate-buffered saline (PBS) to prepare a stock solution of 2000 µg/mL and sterilized through a 0.22 µm Millex-GS syringe filter (Cat. No. SLGS033, Merck Millipore, Burlington, MA, USA). Cells were exposed to four working concentrations of SHXXT: 200, 20, 4, and 2 µg/mL, corresponding to the previously defined 10×, 100×, 500×, and 1000× dilution groups, respectively.

Cellular responses to SHXXT treatment were assessed using carboxyfluorescein succinimidyl ester (CFSE) staining, Annexin V/propidium iodide (PI) staining, and MT-1 mitochondrial activity assays. Flow cytometric data were acquired using a BD FACSLyric™ Flow Cytometry System (BD Biosciences, Eysins, Switzerland).

For Annexin V/PI assays (#556547, BD Biosciences), unlabeled Hep3B cells were seeded under identical conditions and treated for 24 h. Cells were harvested and stained according to the manufacturer’s instructions to distinguish viable, apoptotic, and PI-positive cell populations. Samples were analyzed immediately after staining by flow cytometry.

For mitochondrial function analyses, MT-1 reagent (#MT13-10, Dojindo Molecular Technologies, Rockville, MD, USA) was added following 24 h SHXXT exposure and incubated for 30 min according to the manufacturer’s instructions. Fluorescence signals were acquired using the PE channel of the BD FACSLyric™ system and analyzed using FlowJo software (version 10, BD Biosciences, Ashland, OR, USA; https://www.flowjo.com/). Cells exhibiting high PE fluorescence intensity were classified as MT-1 phycoerythrin (PE)-A+ cells, representing metabolically active cell populations. Results were expressed as the percentage of PE-A+ cells across three independent biological replicates.

All experiments were performed using three independent biological replicates. These exploratory assays were intended to provide supportive biological observations regarding SHXXT-induced cellular responses and were not designed to evaluate therapeutic efficacy, dose optimization, or cancer-type selectivity. Because Hep3B cells are derived from hepatocellular carcinoma rather than renal cell carcinoma, these experiments were conducted solely to provide exploratory biological context for the computational framework and were not intended as disease-specific validation of the KIRC-associated predictions.

## 5. Conclusions

This study established a pan-cancer bioinformatics framework that integrates TCM-associated transcriptomic perturbation profiles, cancer transcriptomic datasets, survival analyses, and prognostic alignment scoring to systematically evaluate the potential relevance of SHXXT-associated gene regulation across multiple cancer types. By combining pharmacogenomic perturbation signatures with cancer-specific expression and survival information, the proposed framework provides a novel strategy for prioritizing candidate cancer types for further investigation.

Among the 24 TCGA cancer cohorts analyzed, KIRC consistently achieved the highest prognostic alignment score and retained the top ranking across multiple normalization and sensitivity analyses. Pathway enrichment analyses further identified PI3K–Akt and MAPK signaling pathways as major biological networks associated with the observed prognostic concordance, highlighting several candidate genes that may contribute to the KIRC-associated transcriptional signatures identified in this study.

Importantly, the findings presented here are based on transcriptomic and survival associations and should not be interpreted as evidence of therapeutic efficacy or clinical benefit. Rather, the proposed framework serves as a hypothesis-generating approach for identifying cancer contexts in which SHXXT-associated regulatory signatures exhibit favorable prognostic alignment.

Future studies incorporating KIRC-specific experimental models, including renal carcinoma cell lines, patient-derived systems, animal models, and independent clinical cohorts, will be required to determine whether the computationally identified transcriptional signatures translate into biologically meaningful therapeutic responses. Beyond SHXXT, the analytical framework presented here may be broadly applicable to other traditional Chinese medicine formulations and natural-product-derived therapeutics, thereby facilitating data-driven prioritization of cancer types for future precision oncology research and translational drug discovery.

## Figures and Tables

**Table 1:** Major Bioactive Constituents of SHXXT and Their Relative Abundance in ccTCM.

HERB	Selected Compound (ITCM)	Relative Abundance in Herb (%)	Corresponding Compound in ccTCM
*Coptis chinensis*	berberine	5.3~7.8%	berberine
	coptisine	1.5~2.7%	Tetrahydrocoptisine
	palmatine	1.3~1.9%	palmatine
*Scutellaria* *baicalensis*	baicalin	9.4%~18.0%	baicalin
	wogonoside	1.3%~3.0%	wogonoside
	baicalein	0.18%~3.10%	baicalein
	luteolin	1.1~1.2%	luteolin
	wogonin	0.05%~0.99%	wogonin
*Rhubarb*	catechin	~1.20%	catechin
	Resveratrol-4′-O-(6″-galloyl)glucoside	0.03~1.70%	Resveratrol
	epicatechin	0.01~1.70%	epicatechin
	aloe-emodin	0.02~0.80%	aloe-emodin
	chrysophanol	0.01~0.17%	chrysophanol
	rhein	0.01~0.10%	rhein
	emodin	0.01~0.07%	emodin

**Table 2:** Summary of TCGA cancer cohorts included in the pan-cancer analysis.

TCGA_ID	Cancer	T	N
BLCA	Bladder urothelial carcinoma	412	19
BRCA	Breast carcinoma	1111	113
CESC	Cervical Squamous Cell Carcinoma and Endocervical Adenocarcinoma	304	3
CHOL	Cholangiocarcinoma	35	9
COAD	Colon Adenocarcinoma	481	41
ESCA	Esophageal carcinoma	184	13
GBM	Glioblastoma multiforme	157	5
HNSC	Head and Neck Squamous Cell Carcinoma	520	44
KICH	Kidney Chromophobe	66	25
KIRC	Kidney renal clear cell carcinoma	541	72
KIRP	Kidney renal papillary	290	32
LIHC	Liver hepatocellular carcinoma	371	50
LUAD	Lung adenocarcinoma	539	59
LUSC	Lung squamous cell carcinoma	502	51
PAAD	Pancreatic adenocarcinoma	178	4
PCPG	Pheochromocytoma and paraganglioma	179	3
PRAD	Prostate adenocarcinoma	501	52
READ	Rectum Adenocarcinoma	166	10
SARC	Sarcoma	259	2
SKCM	Skin cutaneous melanoma	103	1
STAD	Stomach adenocarcinoma	412	36
THCA	Thyroid carcinoma	505	59
THYM	Thymoma	120	2
UCEC	Uterine corpus endometrioid carcinoma	553	35

Abbreviations: T, primary tumor samples; N, solid tissue normal samples. Clinical and transcriptomic data were obtained from TCGA and used for differential expression and survival analyses.

**Table 3:** Pan-cancer prognostic alignment scores associated with SHXXT-regulated genes.

Cancer Type	Total Prognostic Score	Favorable Alignment Score	Unfavorable Alignment Score
KIRC	2358	13,074	−10,716
UCEC	466	1918	−1452
BRCA	463	1409	−946
GBM	443	2135	−1692
CHOL	252	2215	−1963
BLCA	241	1389	−1148
THCA	193	726	−533
LIHC	174	4700	−4526
LUAD	162	1690	−1528
PCPG	124	1430	−1306
CESC	115	1403	−1288
KIRP	94	1440	−1346
READ	49	1415	−1366
HNSC	43	1544	−1501
SKCM	16	21	−5
LUSC	9	1714	−1705
THYM	5	40	−35
PAAD	−25	217	−242
PRAD	−33	590	−623
ESCA	−58	874	−932
COAD	−129	1241	−1370
SARC	−166	228	−394
KICH	−247	881	−1128
STAD	−259	766	−1025

**Table 4:** Representative cancer-associated genes identified in the four prognostic regulatory groups of KIRC.

UG					UB				
Ensg_Id	Gene	Oncogene	TSG	Pathway Count (138)	Ensg_Id	Gene	Oncogene	TSG	Pathway Count (56)
ENSG00000100030	MAPK1	Yes	No	105	ENSG00000102882	MAPK3	Yes	No	49
ENSG00000051382	PIK3CB	Yes	No	100	ENSG00000142208	AKT1	Yes	No	45
ENSG00000121879	PIK3CA	Yes	No	100	ENSG00000141736	ERBB2	Yes	No	17
ENSG00000145675	PIK3R1	No	Yes	99	ENSG00000096968	JAK2	Yes	No	17
ENSG00000133703	KRAS	Yes	No	84	ENSG00000197943	PLCG2	Yes	No	17
ENSG00000132155	RAF1	Yes	No	83	ENSG00000162434	JAK1	Yes	Yes	16
ENSG00000213281	NRAS	Yes	No	81	ENSG00000067560	RHOA	Yes	No	15
ENSG00000115904	SOS1	Yes	No	48	ENSG00000106799	TGFBR1	No	Yes	15
ENSG00000198793	MTOR	Yes	No	46	ENSG00000173757	STAT5B	Yes	No	15
ENSG00000082701	GSK3B	Yes	No	45	ENSG00000149269	PAK1	Yes	No	14
ENSG00000110092	CCND1	Yes	No	45	ENSG00000100311	PDGFB	Yes	No	12
ENSG00000157764	BRAF	Yes	No	44	ENSG00000066468	FGFR2	Yes	No	11
ENSG00000124762	CDKN1A	No	Yes	41	ENSG00000111276	CDKN1B	No	Yes	11
ENSG00000078061	ARAF	Yes	No	36	ENSG00000039068	CDH1	No	Yes	10
ENSG00000171791	BCL2	Yes	No	34	ENSG00000142166	IFNAR1	No	No	10
ENSG00000070831	CDC42	Yes	No	32	ENSG00000068078	FGFR3	Yes	No	10
ENSG00000168036	CTNNB1	Yes	No	32	ENSG00000186350	RXRA	No	No	10
ENSG00000156052	GNAQ	Yes	No	31	ENSG00000160293	VAV2	Yes	No	9
ENSG00000140443	IGF1R	Yes	No	30	ENSG00000078369	GNB1	Yes	No	7
ENSG00000118260	CREB1	Yes	No	30	ENSG00000044115	CTNNA1	No	Yes	6
**DG**					**DB**				
**E** **nsg_** **I** **d**	**Gene**	**Oncogene**	**TSG**	**Pathway Count (37)**	**E** **nsg_** **I** **d**	**Gene**	**Oncogene**	**TSG**	**Pathway Count (12)**
ENSG00000105647	PIK3R2	No	Yes	31	ENSG00000127191	TRAF2	No	No	9
ENSG00000087088	BAX	No	No	22	ENSG00000100906	NFKBIA	No	Yes	8
ENSG00000128340	RAC2	Yes	No	12	ENSG00000023445	BIRC3	Yes	Yes	6
ENSG00000105173	CCNE1	Yes	No	12	ENSG00000006062	MAP3K14	Yes	No	6
ENSG00000170581	STAT2	No	No	11	ENSG00000136997	MYC	Yes	No	6
ENSG00000134574	DDB2	No	Yes	11	ENSG00000140464	PML	No	Yes	4
ENSG00000204525	HLA-C	No	Yes	11	ENSG00000184371	CSF1	No	No	3
ENSG00000206503	HLA-A	No	Yes	11	ENSG00000118503	TNFAIP3	No	Yes	3
ENSG00000234745	HLA-B	No	Yes	11	ENSG00000010671	BTK	Yes	No	3
ENSG00000101096	NFATC2	No	No	11	ENSG00000204267	TAP2	No	No	3
ENSG00000112715	VEGFA	Yes	No	10	ENSG00000168394	TAP1	No	No	3
ENSG00000105976	MET	Yes	No	10	ENSG00000175197	DDIT3	No	No	3
ENSG00000263528	IKBKE	Yes	No	9	ENSG00000146648	EGFR	Yes	No	3
ENSG00000182578	CSF1R	No	No	7	ENSG00000159216	RUNX1	No	Yes	3
ENSG00000160271	RALGDS	No	No	5	ENSG00000137193	PIM1	No	No	2
ENSG00000126458	RRAS	Yes	No	5	ENSG00000139618	BRCA2	No	Yes	2
ENSG00000105327	BBC3	No	Yes	4	ENSG00000069399	BCL3	Yes	No	1
ENSG00000121966	CXCR4	Yes	No	4	ENSG00000125347	IRF1	No	Yes	1
ENSG00000197903	H2BC12	No	No	4	ENSG00000146232	NFKBIE	No	No	1
ENSG00000274641	H2BC17	No	No	4	ENSG00000120875	DUSP4	No	Yes	1

UG and DG represent concordant regulatory groups aligned with favorable prognostic directions, whereas UB and DB represent discordant regulatory groups.

## Data Availability

The original contributions presented in this study are included in the article and Supplementary Materials. The cancer transcriptomic and clinical data analyzed in this study are publicly available from The Cancer Genome Atlas (TCGA) at https://portal.gdc.cancer.gov/ (accessed on 28 January 2024). Gene expression perturbation data for SHXXT active compounds were obtained from the Integrated Traditional Chinese Medicine (ITCM) database. The analytical code used for survival analysis, prognostic scoring and also further inquiries can be directed to the corresponding author upon reasonable request.

## Supplementary Materials

The following supporting information can be downloaded at: https://www.mdpi.com/article/10.3390/ph19060936/s1, Figure S1: Representative flow cytometry plots of MT-1 staining in Hep3B cells treated with SHXXT for 24 h; Table S1: Score normalization analysis across 24 TCGA cancer types; Table S2. Sensitivity analysis under alternative weighting schemes (non-significant: moderate HR: strong HR); Table S3. MT-1 PE-A+ cell percentages (%) from three independent biological replicates (Hep3B cells, SHXXT treatment, 24 h).

## Abbreviations

TCM	Traditional Chinese Medicine
SHXXT	San-Huang-Xie-Xin-Tang
DEGs	Differentially Expressed Genes
HR	Hazard Ratio
TCGA	The Cancer Genome Atlas
ccTCM	Chinese Compound Medicine Database
ITCM	Integrated Traditional Chinese Medicine Database
