# Supplementary material for: Pan-Cancer Bioinformatics-Guided Evaluation of San-Huang-Xie-Xin-Tang Identifies Kidney Renal Clear Cell Carcinoma as a Potentially Responsive Cancer Type
Source: Pharmaceuticals (Basel). 2026 Jun 14;19(6):936. doi: 10.3390/ph19060936 (PMC13304722; doi:10.3390/ph19060936)

Supplementary Figure S1

Representative flow cytometry plots of MT-1 staining in Hep3B cells treated with SHXXT for 24 hours. Cells were analyzed using the BD FACSLyric™ Flow Cytometry System (PE channel). High PE-A+ fluorescence indicates intact mitochondrial membrane potential; low PE-A+ fluorescence indicates mitochondrial dysfunction. Concentrations shown: D0 blank, D1 control, 2 μg/mL (1000×), 4 μg/mL (500×), 20 μg/mL (100×), and 200 μg/mL (10×).

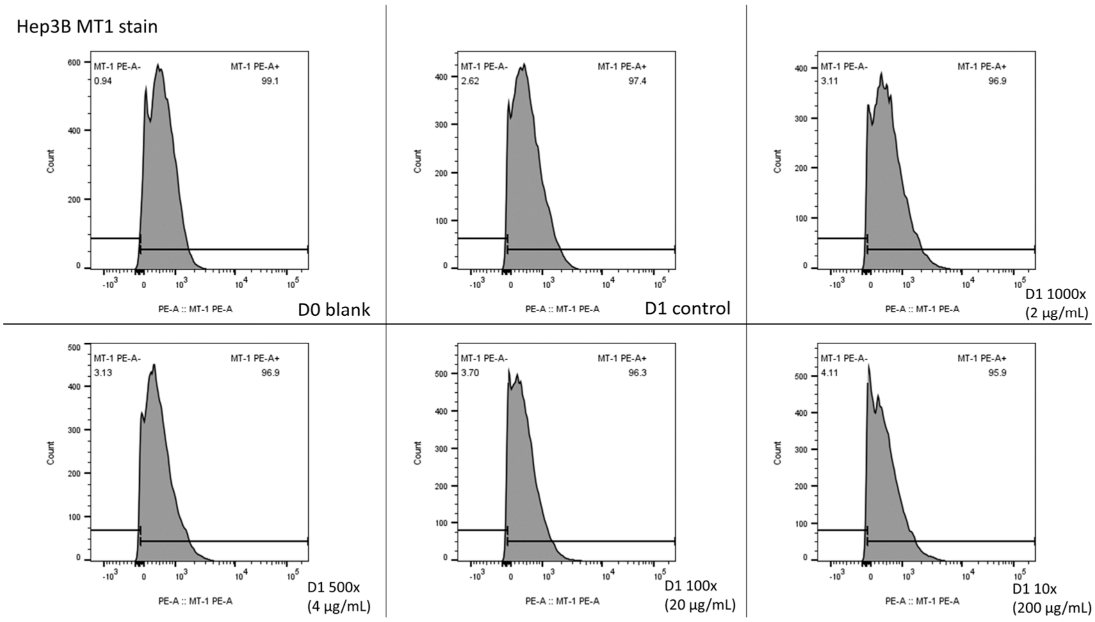

Supplement: Supplementary file 1 [file pharmaceuticals-19-00936-s001.zip › Supplementary_Figures.pdf]
