# Supplementary material for: Pan-Cancer Bioinformatics-Guided Evaluation of San-Huang-Xie-Xin-Tang Identifies Kidney Renal Clear Cell Carcinoma as a Potentially Responsive Cancer Type
Source: Pharmaceuticals (Basel). 2026 Jun 14;19(6):936. doi: 10.3390/ph19060936 (PMC13304722; doi:10.3390/ph19060936)
Supplement: Supplementary file 1 [file pharmaceuticals-19-00936-s001.zip › Supplementary_Tables.pdf]

Table S1. Score normalization analysis across 24 TCGA cancer types.

**Cancer**	**n_tumor**	**n_sig_genes**	**total_score**	**rank (total)**	**score/sig_gene**	**rank (per gene)**	**score/100samples**	**rank (per sample)**	combined rank
KIRC	541	7287	2358	1	0.32359	2	435.8595	2	5
UCEC	553	3213	466	2	0.145036	5	84.26763	4	11
BRCA	1111	2355	463	3	0.196603	3	41.67417	8	14
GBM	157	3827	443	4	0.115756	6	282.1656	3	13
CHOL	35	4178	252	5	0.060316	10	720	1	16
BLCA	412	2392	241	6	0.100753	7	58.49515	6	19
THCA	505	1259	193	7	0.153296	4	38.21782	9	20
LIHC	371	4115	174	8	0.042284	13	46.90027	7	28
LUAD	539	2582	162	9	0.062742	9	30.05566	12	30
PCPG	179	2736	124	10	0.045322	11	69.27374	5	26
CESC	304	2691	115	11	0.042735	12	37.82895	10	33
KIRP	290	2516	94	12	0.037361	14	32.41379	11	37
READ	166	2781	49	13	0.01762	15	29.51807	13	41
HNSC	520	3020	43	14	0.014238	16	8.269231	15	45
SKCM	103	26	16	15	0.615385	1	15.53398	14	30
LUSC	502	3419	9	16	0.002632	17	1.792829	17	50
THYM	120	75	5	17	0.066667	8	4.166667	16	41
PAAD	178	443	-25	18	-0.05643	21	-14.0449	19	58
PRAD	501	1213	-33	19	-0.02721	18	-6.58683	18	55
ESCA	184	1806	-58	20	-0.03212	19	-31.5217	21	60
COAD	481	2611	-129	21	-0.04941	20	-26.8191	20	61
SARC	259	615	-166	22	-0.26992	24	-64.0927	23	69
KICH	66	2009	-247	23	-0.12295	22	-374.242	24	69
STAD	412	1791	-259	24	-0.14461	23	-62.8641	22	69

Sgene: total prognostic score / n_sig_genes (FDR < 0.05 in survival or DEG analysis). Ssample: total prognostic score / n_tumor × 100. KIRC ranked first overall (rank sum = 5) across all three normalization strategies (total score rank, score per significant gene rank, and score per 100 tumor samples rank), confirming the robustness of the prioritization framework. Cancer types with very limited normal sample sizes (e.g., SKCM, CHOL) may show inflated normalized scores and should be interpreted with caution.

Table S2. Sensitivity analysis under alternative weighting schemes (non-significant : moderate HR: strong HR).

Cancer	rank (1:2:4)	score (1:2:4)	rank (0:1:2)	score (0:1:2)	rank (1:2:3)	score (1:2:3)	rank (0:2:4)	score (0:2:4)	rank (1:3:9)	score (1:3:9)
KIRC	1	2358	1	1146	1	1916	1	2292	1	4830
BRCA	3	463	4	0	3	463	4	0	2	463
BLCA	6	241	3	10	6	236	3	20	5	266
CHOL	5	252	6	0	5	252	6	0	6	252
LUAD	9	162	2	30	9	147	2	60	7	237
GBM	4	443	9	0	4	443	9	0	4	443
CESC	11	115	5	0	11	115	5	0	10	115
UCEC	2	466	22	-2	2	467	22	-4	3	461
PCPG	10	124	14	0	10	124	14	0	9	124
THCA	7	193	19	0	8	193	19	0	8	193
HNSC	14	43	10	0	14	43	10	0	14	43
READ	13	49	16	0	13	49	16	0	12	49
LUSC	16	9	12	0	16	9	12	0	16	9
ESCA	20	-58	8	0	20	-58	8	0	20	-58
LIHC	8	174	24	-49	7	200	24	-98	13	47
COAD	21	-129	7	0	21	-129	7	0	21	-129
SKCM	15	16	17	0	15	16	17	0	15	16
PAAD	18	-25	13	0	18	-25	13	0	18	-25
KIRP	12	94	23	-6	12	97	23	-12	11	79
PRAD	19	-33	15	0	19	-33	15	0	19	-33
KICH	23	-247	11	0	23	-247	11	0	23	-247
THYM	17	5	20	0	17	5	20	0	17	5
SARC	22	-166	21	-2	22	-165	21	-4	22	-171
STAD	24	-259	18	0	24	-259	18	0	24	-259

Strong HR: HR > 2 or HR < 0.5; moderate HR: 0.5 ≤ HR ≤ 2. KIRC ranked first across all five weighting schemes.

Table S3. MT-1 PE-A+ cell percentages (%) from three independent biological replicates (Hep3B cells, SHXXT treatment, 24h).

Condition	Replicate 1	Replicate 2	Replicate 3	Mean	SD
D0 blank	99.1	99.0	99.0	99.03	0.06
D1 control	97.4	96.3	97.7	97.13	0.74
2 μg/mL (1000×)	96.9	95.5	95.8	96.07	0.74
4 μg/mL (500×)	96.9	93.4	92.2	94.17	2.44
20 μg/mL (100×)	96.3	93.1	90.5	93.30	2.91
200 μg/mL (10×)	95.9	93.0	89.9	92.93	3.00

Statistical comparisons: Welch's t-test. D0 blank vs. D1 200 μg/mL: p = 0.0497 (*). D1 control vs. all treatment groups: p > 0.05 (ns).
